# Secondary Neoplasms in Children with Hodgkin’s Lymphoma Receiving C-MOPP and Radiotherapy: Presentation of Four Cases

**DOI:** 10.4274/tjh.2015.0027

**Published:** 2016-02-17

**Authors:** Sevgi Gözdaşoğlu, Ali Pamir, Emel Ünal, İsmail Haluk Gökçora, Ömer Uluoğlu, Koray Ceyhan, Haluk Deda, Erdoğan Işıkman, Gülsan Yavuz, Nurdan Taçyıldız, Ayhan Çavdar

**Affiliations:** 1 Ankara University Faculty of Medicine, Department of Pediatric Hematology and Oncology, Ankara, Turkey; 2 Ankara University Faculty of Medicine, Department of Pediatric Surgery, Ankara, Turkey; 3 Gazi University Faculty of Medicine, Department of Pathology, Ankara, Turkey; 4 Ankara University Faculty of Medicine, Department of Pathology, Ankara, Turkey; 5 TOBB University of Economics and Technology Hospital, Clinic of Neurosurgery, Ankara, Turkey; 6 Ankara University Faculty of Medicine, Department of Radiotherapy, Ankara, Turkey

**Keywords:** Secondary neoplasms, Chemoradiotherapy, Hodgkin’s lymphoma

## Abstract

Patients who survive Hodgkin lymphoma (HL) are at increased risk of secondary neoplasms (SNs). A wide variety of SNs have been reported, including leukemias, non-Hodgkin’s lymphomas, and solid tumors, specifically breast and thyroid cancers. Herein we report subsequent neoplasms in four patients with HL receiving chemoradiotherapy. It is interesting that three SNs, fibrosarcoma, thyroid carcinoma, and retrobulbar meningioma, were observed in the radiation area in one of our patients. A hypopharyngeal epithelioid malignant peripheral nerve sheath tumor as an unusual secondary malignant neoplasm developed in another patient, while a benign thyroid nodule and invasive ductal breast carcinoma were observed at different times in the female patient. Follicular adenoma of the thyroid gland developed in one of our patients.

## Abstract (tr)

Yaşayan Hodgkin lenfomalı olgularda ikincil neoplazilerin gelişme riski yüksektir. Lösemiler, Hodgkin-dışı malign lenfomalar ve solid tümörler özellikle meme ve tiroid kanserlerini içeren çok çeşitli ikincil malign neoplaziler raporlanmıştır. Burada kemoradyoterapi alan Hodgkin lenfomalı dört olguda gelişen ikincil neoplaziler sunulmuştur. İlginç olarak hastalarımızdan birinde ışın alanında üç ikincil neoplazm; fibrosarkom, tiroid karsinom ve retrobulber meningiom, diğer bir hastamızda sıradışı ikincil malign neoplazm olarak hipofaringeal epitelyal malign periferik sinir kılıfı tümörü gelişirken kız hastada farklı zamanlarda benign tiroid nodülü ve invaziv duktal meme kanseri saptandı. Bir hastamızda ise, tiroidde folliküler adenoma gelişti.

## INTRODUCTION

Developments in chemoradiotherapy have enabled most patients with Hodgkin’s lymphoma (HL) to be cured. However, the long-term effects of the treatment include an increased risk of secondary neoplasms (SNs). SNs are defined as histologically distinct neoplasms developing at least 2 months after the completion of treatment for the primary malignancy [1]. SNs may be benign or malignant in characteristics. The occurrence of SNs following HL has now been recognized as a major problem. Among long-term survivors who received C-MOPP (cyclophosphamide, vincristine, procarbazine, and prednisone) plus radiotherapy (RT), SNs developed only in 4 out of 28 patients. In 3 respective cases, follicular adenoma of the thyroid gland, invasive ductal breast carcinoma, and hypopharyngeal epithelioid malignant peripheral nerve sheath tumor (EMPNST) occurred individually. In another patient, 3 neoplasms, fibrosarcoma, papillary thyroid cancer, and retrobulbar meningioma, were observed subsequently. Herein we report these cases.

## CASE PRESENTATIONS

### Case 1

**Figure 1:**
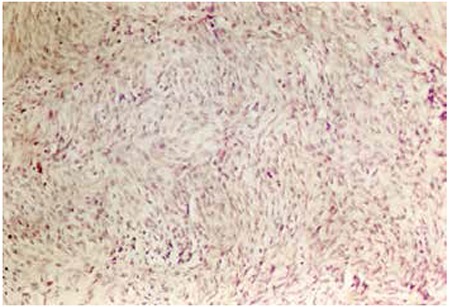
Histopathologic section of fibrosarcoma showing fascicles of spindle-shaped cells (H&E stain, 20x).

A 9-year-old male patient was diagnosed with clinical stage I HL with the mixed cellularity (MC) histopathological subtype and had received local RT of 40 Gy to the neck region. He relapsed 3.5 years later with clinical stage IV disease and was given 6 cycles of C-MOPP and 4 cycles of maintenance C-MOPP, for a total of 10 cycles. A fibrosarcoma developed in the radiation area 8 years following the initial treatment (Figure 1), then followed by papillary thyroid carcinoma diagnosed at 16 years and a right-sided retrobulbar meningioma at 30 years later consecutively. The patient was treated according to his neoplasms and he is alive at the present time.

### Case 2

A 15-year-old male patient was diagnosed with clinical stage I HL with lymphocytic predominance (LP) and received 3 cycles of C-MOPP and 40 Gy RT to the neck region. A thyroid nodule developed 27 years after the initial treatment. Surgical excision was performed and the histopathological diagnosis was follicular adenoma of the thyroid gland. The patient is presently alive with no symptoms.

### Case 3

A 10-year-old female patient was diagnosed with clinical stage II HL of the nodular sclerosis (NS) type and received nitrogen mustard, vinblastine, and local RT (40 Gy) to the neck region. She developed a mediastinal relapse 4 years after the initial treatment and was given cyclophosphamide (CTX), vincristine (VCR), and adriamycin (ADM) (four cycles) and C-MOPP (two cycles), and mediastinal RT at a dose of 37 Gy. She further developed a benign thyroid nodule 14 years later, which was excised. An invasive ductal carcinoma appeared in her left breast 30 years after the initial chemoradiotherapy. After the diagnosis of the breast carcinoma, the patient did not return and was lost to follow-up.

### Case 4

**Figure 2:**
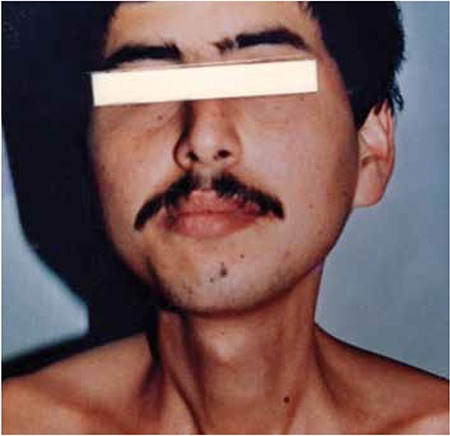
Appearance of swan-like neck.

**Figure 3:**
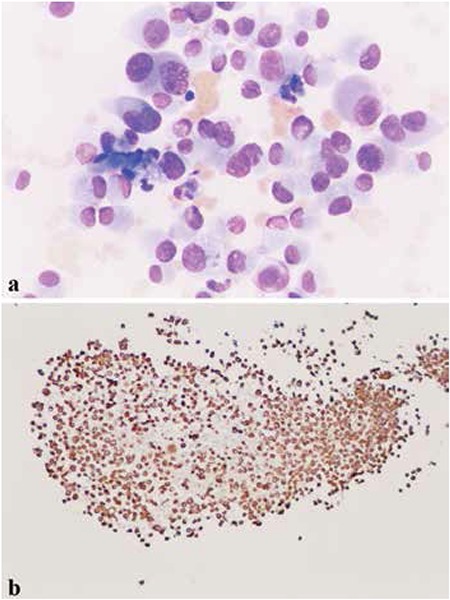
Epithelioid malignant peripheral nerve sheath tumor cells with May Grunwald-Giemsa and diaminobenzidine stainings. a) Binucleated tumor cells and cytoplasmic perinuclear small vacuoles (May Grunwald-Giemsa stain, 200x). b) Diffuse nuclear and cytoplasmic S-100 protein positivity (Diaminobenzidine stain, 40x).

A 13-year-old male patient was diagnosed with clinical stage III HL with MC histopathology and he received 40 Gy RT to the neck region and C-MOPP plus maintenance C-MOPP (a total of 10 cycles). A swan-like neck developed 12 years after the treatment (Figure 2). An EMPNST (Figure 3) developed 30 years after the initial treatment. Although the patient received four cycles of iphosphamide and ADM combination chemotherapy, he died with progression of his malignant disease 6 months after diagnosis.

## DISCUSSION

The incidence of SNs has been extensively investigated in patients treated for HL. The observed number of SNs was 3.8 times that expected among patients treated with MOPP only, 3.2 times that expected among those treated with extended field or total nodal irradiation only, and 23.0 times that expected among those treated with MOPP and extended field or total nodal irradiation [2]. Approximately 25% of the mortality after treatment for HL is believed to be due to SNs [3]. A wide variety of SNs have been reported, including leukemias, non-Hodgkin’s lymphomas, and solid tumors, specifically breast and thyroid cancers. Breast cancer was the most common solid tumor with an estimated actuarial incidence of 35% in women by 40 years of age [4]. In one article, these SNs occurred from 3 months to 21 years after the diagnosis of HL, with leukemias having a median latent period of 5.5 years and solid tumors 9.5 years from diagnosis [5].

Age at treatment has a major effect on the risk of SNs after therapy for HL. A cohort group of 5519 patients with HL treated during 1963-1993 was evaluated and followed for SNs, and it was found that 322 SNs occurred. Relative risks of solid cancers and of leukemia increased significantly with younger age at first treatment [6].

On the other hand, in addition to chemoradiotherapy, genetic predisposing factors such as Li-Fraumeni syndrome, neurofibromatosis, and genetic retinoblastoma further enhance the potential for developing SNs. Genetic susceptibility may play an aggravating role [7].

Thirty-nine children with previously untreated HL were treated with MOPP and RT between 1970 and 1984 in our department. The median age was 10 years (range: 3 to 15 years); 29 were males and 10 were females. The majority of the patients were at stage III-IV with a predominance of the MC histopathological subtype. Twenty-four patients received C-MOPP combination chemotherapy (10-12 cycles), whereas 14 patients were given sandwich therapy of 3 C-MOPP plus EF RT 40 Gy plus 3 C-MOPP, and one case was treated with CTX, VCR, and ADM (4 cycles) and C-MOPP (2 cycles) plus 38 Gy mediastinal RT. Eleven of the 39 patients could not be followed, while 28 patients had a complete evaluation with a median follow-up period of 234 months. Among long-term survivors, SNs occurred only in 4 out of 28 patients: follicular adenoma of the thyroid gland, invasive ductal breast carcinoma, and hypopharyngeal EMPNST, and, in one case, three neoplasms, fibrosarcoma, papillary thyroid cancer, and retrobulbar meningioma, were observed subsequently. All of these SNs developed in the radiation areas. The patients’ details are given in Table 1.

Radiation-related solid SNs account for 80% of all SNs and demonstrate a strong relationship with RT. The risk of these solid tumors increases with the total dose of radiation, exposure at a younger age, and longer follow-up after RT [1].

Radiation-induced fibrosarcoma occurred at random intervals from 3 to 38 years after irradiation, usually after high dosages [8]. Fibrosarcoma developed 8 years after the initial treatment in the radiation area in Case 1. Fibrosarcoma was observed as the first SN in our experience with HL [9].

In our patients, thyroid abnormalities occurred in 50% of the patients who received 40 Gy to the neck region. All patients without irradiation to the neck region showed normal thyroid function [10]. Radiation therapy at a young age is the major risk factor for the development of secondary thyroid cancers. The risk has been reported to be 18-fold that of the general population [1]. In Case 1, papillary thyroid cancer developed 16 years after 40 Gy radiation to the neck. Total thyroidectomy was performed and hormonal replacement treatment was given. In addition to these two malignancies, a right-sided retrobulbar meningioma developed 30 years after the initial RT. It is interesting that these 3 SNs, fibrosarcoma, papillary thyroid cancer, and meningioma, were observed consecutively in the radiation areas of the patient and all 3 tumors were completely resected.

Benign thyroid lesions including follicular adenoma were also reported [11]. Follicular adenoma developed 27 years after the initial treatment in Case 2.

Best et al. identified two variants at chromosome 6q21 associated with decreased basal PRDM1 expression and impaired induction of PRDM1 by radiation exposure [12]. PRDM1 encodes a zinc finger transcriptional repressor involved in a variety of cellular processes including proliferation, differentiation, and apoptosis. Loss of heterozygosity at chromosome 6q was found to be significantly more common in breast cancers following RT for HL than in sporadic breast cancers (42% vs. 10%) [12].

The risk of breast cancer is high among women treated with RT for childhood HL. Excess risk has been reported in female survivors treated with high-dose, extended-volume radiation at age 30 years or younger. In patients treated with chest RT before 16 years of age, the cumulative incidence approaches 20% by age 45 years. Radiation-associated breast cancer has been reported to have more adverse clinicopathological features [1]. Our patient (case 3) was treated with 40 Gy to the neck region at the first admission and 38 Gy to the mediastinum for relapse in addition to chemotherapy.

Case 4 was treated with 40 Gy RT to the neck region and a total of 10 cycles of C-MOPP. A swan-like neck developed in the patient 12 years after the initial treatment and a hypopharyngeal EMPNST developed in the irradiated area 30 years after RT [13]. The epithelioid variant is an unusual form of malignant peripheral nerve sheath tumors with poor prognosis and represents approximately 5% of malignant peripheral nerve sheath tumors [14]. Adamson et al. reported two patients with malignant peripheral nerve sheath tumors of the spine after RT for HL, and despite prompt surgical resection in the patients, the tumors exhibited aggressive behavior [15]. EMPNST is uncommon but an important fatal complication of RT.

There is increasing evidence that RT doses used in the past were higher than necessary. Children receiving RT have an increased risk of developing serious complications such as pulmonary or cardiac toxicity and other cancers later in life. Hence, the new concept is the use of involved side RT and involved node RT. The goal of this new concept is to reduce both treatment volume and treatment dose while maintaining efficacy and minimizing acute and late sequelae [16,17].

## CONCLUSION

All children who have received these treatments remain at risk and continued surveillance is warranted. Treatment alterations have potentially decreased the future appearance of SNs. Monitoring for the detection of late effects in adult survivors of childhood cancer necessitates good collaboration between pediatric and adult oncology units.

## Ethics

Informed Consent: It was taken.

## Figures and Tables

**Table 1:**
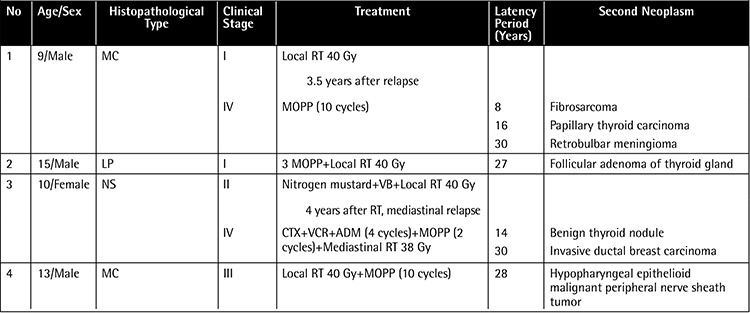
Characteristics of the patients with secondary neoplasias.
